# Neurologic Medication Costs in a Direct-to-Consumer Pharmacy vs Commercial Insurance Plans

**DOI:** 10.1001/jamanetworkopen.2025.27476

**Published:** 2025-08-19

**Authors:** Amanda V. Gusovsky Chevalier, Chun Chieh Lin, Kevin Kerber, Evan L. Reynolds, Brian C. Callaghan, James F. Burke

**Affiliations:** 1 Center for the Advancement of Team Science, Analytics, and Systems Thinking in Health Services and Implementation Science Research (CATALYST), College of Medicine, The Ohio State University, Columbus; 2 Division of General Internal Medicine, College of Medicine, The Ohio State University, Columbus; 3 Department of Neurology, Division of Health Services Research, Wexner Medical Center, The Ohio State University, Columbus; 4 Department of Epidemiology and Biostatistics, Michigan State University, East Lansing; 5 Department of Neurology, University of Michigan, Ann Arbor

## Abstract

**Importance:**

Given increasing costs of neurologic medications, there is a need to explore alternative options to minimize medication spending.

**Objective:**

To compare 2024 neurologic medication costs from commercial insurance plans with those available in the Mark Cuban Cost Plus Drug Company, a direct-to-consumer pharmacy.

**Design, Setting, and Participants:**

This cross-sectional study compared medication costs through a direct-to-consumer pharmacy with model-estimated 2024 costs from commercial and Medicare supplemental databases. Commercial insurance plan costs from 2012 to 2021 were used. The direct-to-consumer pricing for each medication was obtained from the website on December 10, 2024.

**Main Outcomes and Measures:**

The difference between direct-to-consumer and overall commercial insurance plan annual out-of-pocket (OOP) costs and total costs were calculated. Total and OOP costs were separately compared between direct-to-consumer and generic-only commercial prices. Aggregate cost differences were presented for OOP and total costs.

**Results:**

Among 79 neurologic medications considered, 33 medications (42%) available for purchase through the direct-to-consumer pharmacy were studied. The direct-to-consumer pharmacy OOP costs were 75% higher and total costs were 413% lower than commercial pharmacies. Among observed medications, teriflunomide (−40%) and droxidopa (−18%) had lower OOP and total costs in the direct-to-consumer pharmacy, with no other medications having lower OOP costs in the direct-to-consumer pharmacy. In generic-only costs, no direct-to-consumer pharmacy medications had lower OOP annual cost. In total costs, 55% of the direct-to-consumer pharmacy medications had lower total annual cost. Teriflunomide (−5600%), droxidopa (−4800%), and dimethyl fumarate (−1900%) had the largest percentage reduction in the direct-to-consumer pharmacy. In aggregate, if all commercial prescriptions were filled in a direct-to-consumer pharmacy, the aggregate OOP expenditures would increase by $82 million.

**Conclusions and Relevance:**

Practitioners should be aware of options such as direct-to-consumer pharmacies because they can often deliver similar OOP costs for uninsured patients to conventional pharmacies in commercial insurance plans. If pharmacies with direct-to-consumer pharmacy cost structures were widely used, total prescriptions may be markedly reduced.

## Key Points

**Question:**

What is the difference in neurologic medication costs between a direct-to-consumer pharmacy and commercial insurance plans?

**Findings:**

In this cross-sectional study of 33 medications available for purchase through a direct-to-consumer pharmacy, the direct-to-consumer pharmacy out-of-pocket costs were 75% higher and total costs were 413% lower than commercial pharmacies for neurologic medications.

**Meaning:**

These results suggest that a direct-to-consumer pharmacy has the potential to reduce total prescription drug medication costs and offers similar out-of-pocket costs for uninsured patients to conventional pharmacies in commercial insurance plans.

## Teaser

This cross-sectional study compares 2024 costs for neurologic medications between commercial insurance plans and an online, generic-only, direct-to-consumer plan.

## Introduction

Neurologic medications are costly and continue to increase over time,^1^ resulting in financial burden to patients and the health care system. For multiple sclerosis (MS) medications, for example, out-of-pocket (OOP) costs increased by 217% from 2012 to 2021.^2^ The reason for this increase in prescription drug costs is due to many factors, including high-priced new-to-market medications,^2^ the lack of regulation or effective negotiation on pricing, and multistakeholder pricing strategies (ie, arrangements between pharmaceutical companies and pharmacy benefit managers).^3^ Given the increasing costs of neurologic medications, there is a need to explore alternative options to minimize medication spending.

A direct-to-consumer model could mitigate OOP medication costs by retailing to patients directly. This model bypasses the need for insurance payers and pharmacy benefit managers, thereby cutting out a middleperson. Furthermore, the direct-to-consumer pharmacy in this study mainly carries generic medications. This study aimed to compare 2024 neurologic medication costs from commercial insurance plans with that in a direct-to-consumer pharmacy to discern whether there is potential cost savings associated with using a direct-to-consumer pharmacy.

## Methods

We performed a retrospective cross-sectional study comparing the Mark Cuban Cost Plus Drug Company, a direct-to-consumer pharmacy that launched in 2022,^4^ with commercial insurance plan costs of neurologic medications with previously published cost data.^2,5^ The Ohio State University institutional review board deemed this study exempt and individual patient consent was not required because data were deidentified in compliance with the Health Insurance Portability and Accountability Act. The study followed the Strengthening the Reporting of Observational Studies in Epidemiology (STROBE) reporting guideline.

### Commercial Insurance Plan Costs

Commercial insurance plan costs from 2012 to 2021 were previously published.^2,5^ Briefly, total annual commercial insurance plan costs were observed in Merative MarketScan commercial and Medicare supplemental databases to observe patients who had an outpatient neurologist visit with a diagnosis for epilepsy, MS, Parkinson disease, peripheral neuropathy, dementia or Alzheimer disease, amyotrophic lateral sclerosis, transthyretin amyloidosis, Duchenne muscular dystrophy, Huntington disease, myasthenia gravis, migraine or headache, orthostatic hypotension, tardive dyskinesia, or spinal muscular atrophy and had a neurologic medication^2,6^ prescribed within the following 12 months of first diagnosis. Merative MarketScan databases are commercially available and available for purchase. Medications were selected using American Academy of Neurology evidence-based guidelines and systematic reviews to identify common neurologic medications.^2,5^ Annual OOP (sum of copayment, coinsurance, and deductible charges) and total (sum of all charges on the claim) costs were calculated by first summing patient-level cost for each medication, and then for each medication the population-level mean was calculated. Generic-only commercial insurance plan costs were calculated similarly by excluding patients by year with 1 or more brand-name medication fill. Fingolimod and teriflunomide were excluded from the generic-only costs because their generic introduction was after the study period.^6,7^ All conditions and medications from previously described commercial insurance cost analyses on 14 neurologic conditions^2,5^ that were available in this study’s direct-to-consumer pharmacy were included.

### Direct-to-Consumer Pharmacy Costs

The direct-to-consumer pharmacy pricing for each medication was obtained from the website^8^ on December 10, 2024. The direct-to-consumer pharmacy price for a 3-month supply was calculated for each medication by adding the price of the medication to the associated fees (manufacturing, markup, pharmacy labor, and shipping^8^) and then multiplying by 4 to estimate annual 2024 cost. All direct-to-consumer pharmacy costs are OOP due to the direct-to-consumer model.

Cost trends typically decrease sharply for medications in the first several years after generic introduction, whereas medications that have been generic for many years have relatively stable costs.^2^ Conversely, new-to-market medications that maintain market exclusivity enter the market at higher costs than generic alternatives and costs typically increase over time.^2^ The direct-to-consumer pharmacy costs were obtained in 2024, where commercial insurance plan costs were observed from 2012 to 2021. To best estimate 2024 costs while accounting for the influence of generic status and introduction timing on cost, we built 4 linear models to account for price changes over time: (1) generic and branded OOP costs, (2) generic and branded total costs, (3) generic-only OOP costs, and (4) generic-only total costs. The models included all medications that met study criteria on the years of data previously reported,^2^ which were 2012 to 2021. The models’ outcome was log(cost), and the factors were time (years since 2012), whether log(cost) was ever greater than 9 (ie, binary high cost), log(cost in 2021), number of years since the medication became generic,^2^ number of generic firms, and number of other therapies in class. Overall model performance was characterized with *r*^2^ (0.94 for total cost model, 0.88 for OOP model, 0.83 for generic total cost model, and 0.70 for generic OOP model), and calibration was evaluated by examining plots of estimated vs actual total and OOP costs (2012-2021) across 3 groups of medications: never generic, always generic, and became generic. Costs presented were exponentiated and inflation adjusted to 2024 US dollars. Using model-estimated values of costs enabled improved estimates of 12 months of use in 2024 to detect differences in the direct-to-consumer pharmacy. More information about the 4 models is in eAppendixes 1 to 4 in Supplement 1.

### Outcomes

The difference between the direct-to-consumer pharmacy and commercial insurance plan OOP costs and total costs were calculated. Negative values signified that the direct-to-consumer pharmacy cost was lower than commercial insurance plan cost. The aggregate difference between the direct-to-consumer pharmacy and commercial insurance plan OOP and total costs was calculated by multiplying the direct-to-consumer pharmacy and commercial insurance plan OOP and total costs by the respective number of patients observed in the commercial insurance claims database with the medication in 2021 and then subtracting from each other. Similarly, negative values signified that the direct-to-consumer pharmacy cost was lower than commercial insurance plan cost.

### Statistical Analysis

The percentage cost reduction associated with MCCPDC vs commercial insurance plans for each medication was observed for OOP and total costs for all medications and generic-only medications. The average percentage cost reduction associated with MCCPDC across all medications was observed. Analysis was completed using SAS version 9.4 (SAS Institute) and STATA version 18 (StataCorp).

## Results

Among 79 neurologic medications considered, we identified 33 medications (42%) that were available for purchase through the direct-to-consumer pharmacy. The medicines not available in the direct-to-consumer pharmacy were lacosamide, some antipsychotics, deutetrabenazine, interferon beta-1a, interferon beta-1b, gabapentin, pregabalin, tramadol, clonazepam, and other branded medications. Diflunisal, tetrabenazine, droxidopa, methotrexate, and cyclosporine had sample sizes less than 100, and lamotrigine and levetiracetam had the largest sample sizes (both >12 000).

When averaging the difference in costs across all medications observed, the direct-to-consumer pharmacy OOP costs were 75% higher and total costs were 413% lower than commercial insurance plans on average. The medications with the highest 2024 insurance plan annual OOP costs were teriflunomide ($286), droxidopa ($238), fingolimod ($240), dimethyl fumarate ($207), glatiramer acetate ($203), and tetrabenazine ($172). Similarly, the medications with the highest 2024 insurance plan total costs were teriflunomide ($11 739), droxidopa ($11 618), fingolimod ($8394), glatiramer acetate ($6759), dimethyl fumarate ($6698), and tetrabenazine ($5040), with no others above $1000 annually (Table). The same medications had the highest generic-only OOP and total costs except fingolimod and teriflunomide, which were not available in generic form during the study period. The most expensive medications in 2024 in the direct-to-consumer pharmacy were glatiramer acetate ($24 186), fingolimod ($2185), and cyclosporine ($2185), and the rest of the medications examined were less than $635 annually (Table).

**Table.** Neurologic Medication Individual-Level Costs for 1 Year of Prescription in Direct-to-Consumer Pharmacy and Commercial Insurance Plans^a^

Indicated condition and medication	Direct-to-consumer pharmacy cost, 2024 US $	Generic plus branded medications		Generic-only medications	
		OOP cost reduction in direct-to-consumer pharmacy, %	Total cost reduction in direct-to-consumer pharmacy, %	Generic OOP cost reduction in direct-to-consumer pharmacy, %	Generic total cost reduction in direct-to-consumer pharmacy, %
ALS					
Riluzole	177.32	77.01	−102.44^b^	76.50	−156.28^b^
ATTR					
Diflunisal	634.08	97.65	90.08	97.46	88.16
Dementia or AD					
Donepezil	76.00	85.29	52.70	84.67	42.10
Galantamine	148.88	84.90	16.91	84.62	−1.81^b^
Memantine^c^	180.08	87.55	33.16	86.98	18.98
Rivastigmine^c^	113.28	69.71	−123.33^b^	70.06	−157.78^b^
DMD or MG					
Prednisone^c^	84.08	87.83	64.42	88.99	62.87
Epilepsy					
Carbamazepine	154.00	83.31	−1.38^b^	85.38	−8.54^b^
Lamotrigine	245.20	87.99	20.53	89.54	16.25
Levetiracetam	113.36	81.00	−0.43^b^	81.66	−16.59^b^
Topiramate	75.48	55.32	−229.18^b^	61.80	−243.78^b^
Valproate	72.48	69.67	−62.25^b^	70.74	−87.63^b^
HD or TD					
Tetrabenazine^c^	538.40	68.04	−836.08^b^	81.59	−814.31^b^
MG					
Azathioprine	176.28	90.53	57.43	90.72	49.79
Cyclosporine^c^	2184.96	96.96	64.63	97.35	65.92
Methotrexate^c^	124.92	90.51	69.11	91.53	68.30
Mycophenolate	120.84	78.61	−26.74^b^	82.47	−25.75^b^
MS					
Dimethyl fumarate^c^	336.00	38.33	−1893.37^b^	69.42	−1503.33^b^
Fingolimod^c^	2185.20	89.00	−284.11^b^	95.55	−149.87^b^
Glatiramer acetate^c^	24 185.60	99.16	72.05	99.52	72.79
Teriflunomide^c^	205.16	−39.56^b^	−5621.83^b^	28.37^b^	−4589.83^b^
OH					
Droxidopa	238.24	−17.99^b^	−4776.69^b^	33.66^b^	−4357.34^b^
Fludrocortisone	238.76	95.21	84.10	95.37	81.31
Midodrine	89.32	78.25	−10.10^b^	75.64	−50.48^b^
PD					
Carbidopa-levodopa^c^	127.52	64.66	−215.11^b^	65.33	−251.53^b^
Pramipexole	84.48	67.78	−103.61^b^	67.43	−147.05^b^
Rasagiline^c^	279.44	79.61	−120.92^b^	79.07	−164.77^b^
Ropinirole	105.36	77.17	−32.36^b^	76.70	−63.07^b^
PN					
Amitriptyline	121.20	93.11	81.99	93.27	86.38
Duloxetine^c^	100.00	86.57	51.66	87.47	48.00
Nortriptyline	153.60	95.81	90.83	95.86	89.55
Venlafaxine	107.12	89.38	65.92	90.58	64.40
TD or PD					
Amantadine	119.68	71.15	−121.25^b^	72.75	−156.22^b^

Abbreviations: AD, Alzheimer disease; ALS, amyotrophic lateral sclerosis; ATTR, transthyretin amyloidosis; DMD, Duchenne muscular dystrophy; HD, Huntington disease; MG, myasthenia gravis; MS, multiple sclerosis; OH, orthostatic hypotension; OOP, out of pocket; PD, Parkinson disease; PN, peripheral neuropathy; TD, tardive dyskinesia.

^a^ Cost per patient for 1 year of medication fills in commercial insurance plans and in the direct-to-consumer pharmacy and sample size in commercial insurance plans model-estimated 2024 costs from a nationally representative data source (Merative MarketScan commercial claims and encounters databases).

^b^ Commercial insurance plan OOP and total costs if lower than direct-to-consumer pharmacy cost.

^c^ Medications that became generic from 2012 to 2024.

### Annual OOP and Total Cost Differences at the Patient Level

Among the 33 medications observed, 2 (6%) favored the direct-to-consumer pharmacy for having a cheaper OOP annual cost. Teriflunomide (−40%) and droxidopa (−18%) favored the direct-to-consumer pharmacy for having a cheaper cost in the largest value, with no other medications being cheaper in the direct-to-consumer pharmacy in OOP costs (Figure). In generic-only costs, no medications favored the direct-to-consumer pharmacy for having a cheaper OOP annual cost.

**Figure.**
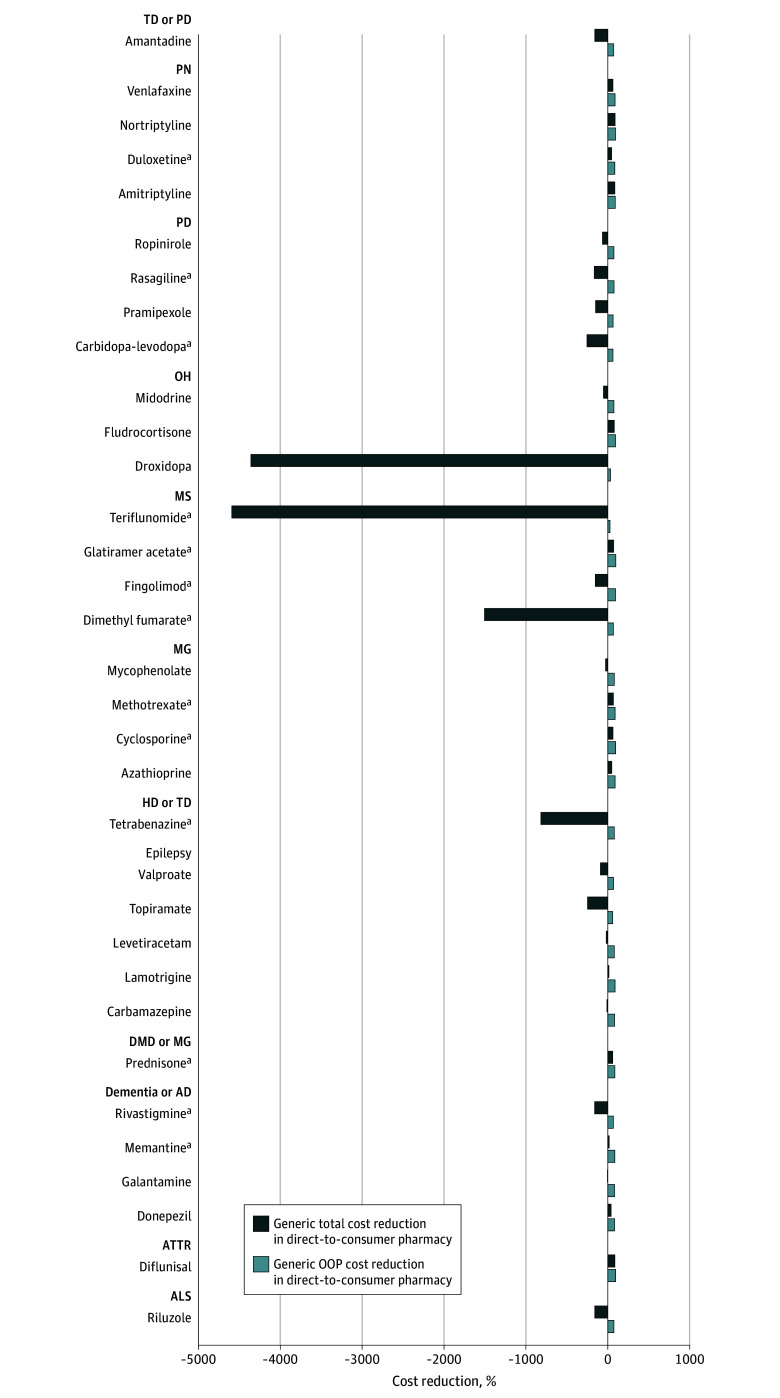
Percentage Difference in Out-of-Pocket and Total Costs Between the Direct-to-Consumer Pharmacy and Commercial Insurance Plans in 2024 in Generic-Only Medication Costs AD indicates Alzheimer disease; ALS, amyotrophic lateral sclerosis; ATTR, transthyretin amyloidosis; DMD, Duchenne muscular dystrophy; HD, Huntington disease; MG, myasthenia gravis; MS, multiple sclerosis; OH, orthostatic hypotension; OOP, out-of-pocket; PD, Parkinson disease; PN, peripheral neuropathy; and TD, tardive dyskinesia. ^a^Medications that became generic from 2012 to 2024.

In total costs, 18 of the 33 medications observed (55%) favored the direct-to-consumer pharmacy for having lower total annual cost. Teriflunomide (−5622%), droxidopa (−4777%), dimethyl fumarate (−1894%), and tetrabenazine (−836%) had the largest percentage reduction in cost between the direct-to-consumer pharmacy and commercial insurance plans, all favoring the direct-to-consumer pharmacy. No medications favored commercial insurance claims by more than 300% annually (Figure). Findings were similar for generic-only total costs.

### Annual OOP and Total Cost Differences at the Aggregate Level

At the aggregate level (eg, medication costs multiplied by number of patients in the commercial insurance claims database with the medication), teriflunomide and droxidopa had savings of −$142 765 and −$2743 associated with the direct-to-consumer pharmacy, respectively. Glatiramer acetate was an outlier in aggregate OOP difference, favoring insurance plans by more than $71 million ($13 million for generic-only costs). Carbidopa-levodopa, venlafaxine, nortriptyline, dimethyl fumarate, valproate, carbamazepine, donepezil, amitriptyline, memantine, duloxetine, levetiracetam, lamotrigine, and fingolimod all favored insurance plans in aggregate OOP costs by more than $100 000. In aggregate, if all commercial prescriptions were filled in a direct-to-consumer pharmacy, the aggregate OOP expenditures would increase by $82 million.

The medications with the largest absolute difference in aggregate total costs between the direct-to-consumer pharmacy and commercial insurance plans were teriflunomide (−$20 287 857), fingolimod (−$11 498 005), and dimethyl fumarate (−$11 139 397). In aggregate, if all commercial prescriptions were filled in a direct-to-consumer pharmacy, the aggregate total expenditures would increase by approximately $9 million.

## Discussion

We found that the direct-to-consumer pharmacy commonly had lower total costs, in some cases much lower, for commonly used generic neurologic medications (except for diflunisal, nortriptyline, and fludrocortisone). However, even when total costs were much lower, this did not translate into lower OOP costs for patients with commercial insurance. There were only 2 medications for which the direct-to-consumer pharmacy offered a lower annual OOP cost and none when compared with generic-only costs. The direct-to-consumer pharmacy may be an important resource, particularly for patients without prescription coverage, because it offers several generic medications for a comparable or cheaper OOP price than commercial insurance plans. However, the direct-to-consumer pharmacy has a somewhat limited formulary—only 42% of commercially available medications were listed at the time of analysis. Although more medications are being added to the site continually, the direct-to-consumer pharmacy will need to be comprehensive in its offerings and offer competitive pricing to provide a viable alternative resource for prescription drug fills for insured patients. Neurologists should be mindful of the existence of this resource and consider whether it may be a valuable alternative for their patients. These data suggest that resources that connect patients and practitioners to cost-saving alternatives may be beneficial.

For most of the neurologic medications observed, the direct-to-consumer pharmacy total cost is estimated to be less than that of commercial insurance plans. The largest aggregate total cost savings were for teriflunomide, fingolimod, and dimethyl fumarate, with aggregate savings estimated at a minimum of $11 million each by using the direct-to-consumer pharmacy instead of commercial insurance plans at a retail pharmacy. These substantial aggregate savings demonstrate the potential of the direct-to-consumer pharmacy to minimize cost expenditures by way of its novel model, thereby alleviating cost burden to the health care system at large. Although it is difficult to precisely specify why and how the direct-to-consumer pharmacy has such a lower total, it is likely that its simpler pricing structure reduces costs compared with the complex commercial status quo involving insurers, pharmacy benefit managers, and pharmacies.

Medications for MS have been the highest-cost,^2,9,10,11^ high-prevalence neurologic medications for more than 20 years and should be prioritized for value optimization given their persistent high prices and high prevalence. The direct-to-consumer pharmacy has 4 MS medications on formulary, 3 of which had markedly reduced total costs compared with insurance plans, with glatiramer acetate the exception. Alternative channels to accessing MS medications may become increasingly important over time because total costs for MS medications have been continually increasing.^2^ The direct-to-consumer pharmacy may be a particularly useful resource for uninsured patients with MS and practitioners to aid in direct-to-patient cost savings. Some large institutions have built relationships with the direct-to-consumer pharmacy.^12^ If a substantial portion of US patients received medications using a price structure similar to that of the direct-to-consumer pharmacy or other online pharmacies, it may pressure commercial insurers and pharmacies to reduce prices through conventional channels to remain competitive.

Two medications had lower OOP costs in the direct-to-consumer pharmacy, but most have somewhat higher OOP costs. The magnitude of the OOP differences between the direct-to-consumer pharmacy and commercial plans, however, were typically not exceptionally large (absolute difference, <$200 in 76% of cases). For patients with unacceptably high OOP costs via commercial insurance, it may be worthwhile to assess whether the direct-to-consumer pharmacy has a less costly option, particularly as new medications become available and costs change in the direct-to-consumer pharmacy.

Existing literature has observed cost differences between the use of the direct-to-consumer pharmacy vs health insurance plans. Kouzy et al^13^ used nationally representative survey data (Medical Expenditure Panel Survey) to examine OOP cost differences for 124 generic medications and found direct-to-consumer pharmacy cost savings associated with less than 12% of the medications. Cortese et al^14^ and Lalani et al^15^ explored the Medicare Part D system-level total cost savings associated with the direct-to-consumer pharmacy and found significant potential savings for Medicare associated with the direct-to-consumer pharmacy prices. These findings are aligned with those of the current study because OOP cost savings were observed in only a few medications examined, for which total cost savings were potentially substantial.

Although the potential cost savings of the direct-to-consumer pharmacy are promising, the future consequences that the direct-to-consumer pharmacy may have on individual patients should be considered. Because all medications are not available through the direct-to-consumer pharmacy, patients may get some medications through traditional pharmacies and others through the direct-to-consumer pharmacy, potentially leading to fragmented care and patient confusion and possibly posing a threat to medication adherence. Furthermore, if patients access some medications through channels such as the direct-to-consumer pharmacy, pharmacists and other practitioners may not see the patient’s full medication regimen, which may lead to potentially harmful outcomes, such as nonadherence or unintended drug-drug interactions. Finding ways to integrate direct-to-consumer pharmacy use into patients’ current health care system interactions would optimize its cost savings while minimizing these potential risks. Furthermore, embedded pharmacy resources connecting practitioners to cost-saving alternatives could be beneficial, including electronic medical record interventions to drive value in prescribing decision-making.

### Limitations

This study has some limitations. Commercial insurance plan costs were from neurologist-prescribed prescriptions, which may differ from those of other practitioner types, so the results of this study should be interpreted as such. We used modeling to estimate 2024 commercial insurance plan costs from real data through 2021, which may be misestimated if insurance plan costs varied in ways for which our model cannot account. Furthermore, we estimated costs for the entire year of 2024, during which medication costs may have fluctuated on a monthly basis. Some medications were not available in the direct-to-consumer pharmacy and therefore were not included in the analyses; however, as more medications become available in the direct-to-consumer pharmacy, analyses should be updated. Next, there is no information in the database regarding discounts or rebates; therefore, these are not accounted for in our analyses. Additionally, the study period (2012-2021) of observed insurance plan costs spans the COVID-19 pandemic. However, 2024 estimated costs account for all the historical data before the pandemic and is therefore our best approximation.

## Conclusions

Generic-only online pharmacies such as the direct-to-consumer pharmacy may be viable alternatives for patients with neurologic conditions to explore cheaper prescription medications, particularly those without prescription coverage. Regulation or effective negotiation on drug pricing and cost-sharing reform may help translate cost savings to individual patients. Other policy solutions to reduce costs for patients are needed. Patients should be encouraged to browse online pharmacies such as the direct-to-consumer pharmacy for potential cost savings and empowered to compare pricing options.

## Notes

Supplement 1. **eAppendix 1.** Out-of-Pocket Costs Predictive Model **eAppendix 2.** Total Costs Predictive Model **eAppendix 3.** Generic-Only Out-of-Pocket Costs Predictive Model **eAppendix 4.** Generic-Only Total Costs Predictive Model

Supplement 2.
Data Sharing Statement
