# Supplementary material for: Neurologic Medication Costs in a Direct-to-Consumer Pharmacy vs Commercial Insurance Plans
Source: JAMA Netw Open. 2025 Aug 19;8(8):e2527476. doi: 10.1001/jamanetworkopen.2025.27476 (PMC12365698; doi:10.1001/jamanetworkopen.2025.27476)
Supplement: Supplement 1. — eAppendix 1. Out-of-Pocket Costs Predictive Model eAppendix 2. Total Costs Predictive Model eAppendix 3. Generic-Only Out-of-Pocket Costs Predictive Model eAppendix 4. Generic-Only Total Costs Predictive Model [file jamanetwopen-e2527476-s001.pdf]

Supplemental Online Content

Gusovsky Chevalier AV, Lin A, Kerber K, Reynolds EL, Callaghan B, Burke JF. Neurologic medication costs in the Mark Cuban Cost Plus Drug Company vs commercial insurance plans. *JAMA Netw Open*. 2025;8(8):e2527476. doi:10.1001/jamanetworkopen.2025.27476

eAppendix 1. Out-of-Pocket Costs Predictive Model

eAppendix 2. Total Costs Predictive Model

eAppendix 3. Generic-Only Out-of-Pocket Costs Predictive Model

eAppendix 4. Generic-Only Total Costs Predictive Model

This supplemental material has been provided by the authors to give readers additional information about their work.

© 2025 Gusovsky Chevalier AV et al. *JAMA Network Open*.

eAppendix 1. Out-of-Pocket Costs Predictive Model

oop-ModelSummary

OLS Regression Results						
**Dep. Variable:**	MEAN_OOP_LOG	R-squared:	0.879			
**Model:**	OLS	Adj. R-squared:	0.877			
**Method:**	Least Squares	F-statistic:	383.8			
**Date:**	Wed	11 Jun 2025	Prob (F-statistic):	4.12E-142		
**Time:**	16:30:11	Log-Likelihood:	-154.31			
**No. Observations:**	324	AIC:	322.6			
**Df Residuals:**	317	BIC:	349.1			
**Df Model:**	6					
**Covariance Type:**	nonrobust					
	coef	std err	t	P>|t|	[0.025	0.975]
**Intercept**	0.0091	0.001	7.172	0.000	0.007	0.012
**TIME**	0.1181	0.016	7.172	0.000	0.086	0.150
**everHighCost**	0.2388	0.115	2.071	0.039	0.012	0.466
**logCost2021**	0.4361	0.027	16.341	0.000	0.384	0.489
**yearsSinceGeneric**	-0.0255	0.007	-3.826	0.000	-0.039	-0.012
**I(yearsSinceGeneric ** 2)**	0.0004	0.000	3.504	0.001	0.000	0.001
**num_gen_firms**	-0.0456	0.015	-2.981	0.003	-0.076	-0.016
**others_in_class**	-0.0212	0.010	-2.165	0.031	-0.041	-0.002
**Omnibus:**	13.493	Durbin-Watson:	0.615			
**Prob(Omnibus):**	0.001	Jarque-Bera (JB):	16.689			
**Skew:**	0.371	Prob(JB):	0.000238			
**Kurtosis:**	3.827	Cond. No.	4.48E+18			
**Notes:**						
**[1] Standard Errors assume that the covariance matrix of the errors is correctly specified.**						
**[2] The smallest eigenvalue is 1.11e-29. This might indicate that there are**						
**strong multicollinearity problems or that the design matrix is singular.**						

© 2025 Gusovsky Chevalier AV et al. *JAMA Network Open*.

eAppendix 2. Total Costs Predictive Model

total-ModelSummary

OLS Regression Results						
**Dep. Variable:**	MEAN_TOTAL_LOG	R-squared:	0.939			
**Model:**	OLS	Adj. R-squared:	0.938			
**Method:**	Least Squares	F-statistic:	699.5			
**Date:**	Wed	11 Jun 2025	Prob (F-statistic):	3.74E-188		
**Time:**	16:28:56	Log-Likelihood:	-220.67			
**No. Observations:**	324	AIC:	457.3			
**Df Residuals:**	316	BIC:	487.6			
**Df Model:**	7					
**Covariance Type:**	nonrobust					
	coef	std err	t	P>|t|	[0.025	0.975]
**Intercept**	2.1189	0.219	9.673	0.000	1.688	2.550
**TIME**	-0.0062	0.010	-0.621	0.535	-0.026	0.014
**everHighCost**	0.5784	0.142	4.075	0.000	0.299	0.858
**logCost2021**	0.7626	0.031	24.245	0.000	0.701	0.824
**yearsSinceGeneric**	-0.0399	0.007	-5.368	0.000	-0.054	-0.025
**I(yearsSinceGeneric ** 2)**	0.0006	0.000	4.050	0.000	0.000	0.001
**num_gen_firms**	-0.0878	0.019	-4.719	0.000	-0.124	-0.051
**others_in_class**	-0.0386	0.012	-3.180	0.002	-0.063	-0.015
**Omnibus:**	21.727	Durbin-Watson:	0.426			
**Prob(Omnibus):**	0.000	Jarque-Bera (JB):	68.441			
**Skew:**	-0.125	Prob(JB):	1.37E-15			
**Kurtosis:**	5.238	Cond. No.	5.37E+03			
**Notes:**						
**[1] Standard Errors assume that the covariance matrix of the errors is correctly specified.**						
**[2] The condition number is large**	5.37e+03. This might indicate that there are					
**strong multicollinearity or other numerical problems.**						

© 2025 Gusovsky Chevalier AV et al. *JAMA Network Open*.

eAppendix 3. Generic-Only Out-of-Pocket Costs Predictive Model

genericOOP-ModelSummary

OLS Regression Results						
**Dep. Variable:**	MEAN_OOP_LOG_G	R-squared:	0.701			
**Model:**	OLS	Adj. R-squared:	0.695			
**Method:**	Least Squares	F-statistic:	109.4			
**Date:**	Wed	11 Jun 2025	Prob (F-statistic):	1.98E-70		
**Time:**	16:30:23	Log-Likelihood:	-184.34			
**No. Observations:**	287	AIC:	382.7			
**Df Residuals:**	280	BIC:	408.3			
**Df Model:**	6					
**Covariance Type:**	nonrobust					
	coef	std err	t	P>|t|	[0.025	0.975]
**Intercept**	0.0115	0.002	7.335	0.000	0.008	0.015
**TIME**	0.1498	0.020	7.335	0.000	0.110	0.190
**everHighCost**	-0.2271	0.149	-1.521	0.129	-0.521	0.067
**logCost2021**	0.3879	0.033	11.868	0.000	0.324	0.452
**yearsSinceGeneric**	-0.0268	0.008	-3.256	0.001	-0.043	-0.011
**I(yearsSinceGeneric ** 2)**	0.0004	0.000	3.086	0.002	0.000	0.001
**num_gen_firms**	-0.0989	0.024	-4.150	0.000	-0.146	-0.052
**others_in_class**	-0.0287	0.013	-2.261	0.025	-0.054	-0.004
**Omnibus:**	74.619	Durbin-Watson:	0.960			
**Prob(Omnibus):**	0.000	Jarque-Bera (JB):	468.752			
**Skew:**	-0.875	Prob(JB):	1.63E-102			
**Kurtosis:**	9.012	Cond. No.	1.63E+18			
**Notes:**						
**[1] Standard Errors assume that the covariance matrix of the errors is correctly specified.**						
**[2] The smallest eigenvalue is 8.38e-29. This might indicate that there are**						
**strong multicollinearity problems or that the design matrix is singular.**						

© 2025 Gusovsky Chevalier AV et al. *JAMA Network Open*.

eAppendix 4. Generic-Only Total Costs Predictive Model

genericTotal-ModelSummary

OLS Regression Results						
**Dep. Variable:**	MEAN_TOTAL_LOG_G	R-squared:	0.830			
**Model:**	OLS	Adj. R-squared:	0.826			
**Method:**	Least Squares	F-statistic:	227.9			
**Date:**	Wed	11 Jun 2025	Prob (F-statistic):	1.23E-104		
**Time:**	16:30:14	Log-Likelihood:	-278.87			
**No. Observations:**	287	AIC:	571.7			
**Df Residuals:**	280	BIC:	597.3			
**Df Model:**	6					
**Covariance Type:**	nonrobust					
	coef	std err	t	P>|t|	[0.025	0.975]
**Intercept**	0.0146	0.002	6.687	0.000	0.010	0.019
**TIME**	0.1899	0.028	6.687	0.000	0.134	0.246
**everHighCost**	0.4514	0.208	2.174	0.031	0.043	0.860
**logCost2021**	0.7243	0.045	15.940	0.000	0.635	0.814
**yearsSinceGeneric**	-0.0295	0.011	-2.570	0.011	-0.052	-0.007
**I(yearsSinceGeneric ** 2)**	0.0003	0.000	1.938	0.054	-5.31E-06	0.001
**num_gen_firms**	-0.1415	0.033	-4.273	0.000	-0.207	-0.076
**others_in_class**	-0.0482	0.018	-2.739	0.007	-0.083	-0.014
**Omnibus:**	144.853	Durbin-Watson:	0.758			
**Prob(Omnibus):**	0.000	Jarque-Bera (JB):	2545.217			
**Skew:**	-1.591	Prob(JB):	0.00			
**Kurtosis:**	17.238	Cond. No.	1.63E+18			
**Notes:**						
**[1] Standard Errors assume that the covariance matrix of the errors is correctly specified.**						
**[2] The smallest eigenvalue is 8.38e-29. This might indicate that there are**						
**strong multicollinearity problems or that the design matrix is singular.**						

© 2025 Gusovsky Chevalier AV et al. *JAMA Network Open*.
