# Supplementary material for: Neurologic Medication Costs in a Direct-to-Consumer Pharmacy vs Commercial Insurance Plans
Source: JAMA Netw Open. 2025 Aug 19;8(8):e2527476. doi: 10.1001/jamanetworkopen.2025.27476 (PMC12365698; doi:10.1001/jamanetworkopen.2025.27476)
Supplement: Supplement 2. — Data Sharing Statement [file jamanetwopen-e2527476-s002.pdf]

Data Sharing Statement

Gusovsky Chevalier. Neurologic Medication Costs in the Mark Cuban Cost Plus Drug Company vs Commercial Insurance Plans. *JAMA Netw Open*. Published August 19, 2025. doi:10.1001/jamanetworkopen.2025.27476

Data
Data available: No

Additional Information
Explanation for why data not available: Prescription pricing data is derived from a private dataset that has limitations on making data available outside of a DUA with the data provider (Marketscan). We could make the Mark Cuban pharmacy data available, though.
